# Immune responses to oligomeric α-synuclein in Parkinson’s disease peripheral blood mononuclear cells

**DOI:** 10.1007/s00415-024-12554-3

**Published:** 2024-07-10

**Authors:** Ana Florencia Vega-Benedetti, Clara Porcedda, Tommaso Ercoli, Giuliana Fusco, Chiara Burgaletto, Rita Pillai, Francesca Palmas, Anna Flavia Cantone, Fabrizio Angius, Paolo Solla, Alfonso De Simone, Giuseppina Cantarella, Cesarina Giallongo, Valeria Sogos, Giovanni Defazio, Anna R. Carta

**Affiliations:** 1 Department of Biomedical Sciences, University of Cagliari, Cagliari, Italy; 2 Department of Medical Sciences and Public Health, University of Cagliari, Cagliari, Italy; 3 Centre for Misfolding Diseases, Department of Chemistry, University of Cambridge, Cambridge, UK; 4 Department of Biomedical and Biotechnological Sciences, University of Catania, Catania, Italy; 5 Center for Research University Services–CeSAR, University of Cagliari, Cagliari, Italy; 6 Department of Medicine, Surgery and Pharmacy, University of Sassari, Sassari, Italy; 7 Department of Pharmacy, University of Naples “Federico II”, 80131 Naples, Italy; 8 Department of Medical, Surgical Sciences and Advanced Technologies G.F. Ingrassia, University of Catania, Catania, Italy; 9 Department of Translational Biomedicine and Neuroscience, Aldo Moro University of Bari, Bari, Italy

**Keywords:** PBMC, monocytes, natural killers, cytokines, constipation, olfaction

## Abstract

Parkinson’s disease displays clinical heterogeneity, presenting with motor and non-motor symptoms. Heterogeneous phenotypes, named brain-first and body-first, may reflect distinct α-synuclein pathology starting either in the central nervous system or in the periphery. The immune system plays a prominent role in the central and peripheral pathology, with misfolded α-synuclein being placed at the intersection between neurodegeneration and inflammation. Here, we characterized the inflammatory profile and immune-phenotype of peripheral blood mononuclear cells (PBMCs) from Parkinson’s disease patients upon stimulation with α-synuclein monomer or oligomer, and investigated relationships of immune parameters with clinical scores of motor and non-motor symptoms. Freshly isolated PBMCs from 21 Parkinson’s disease patients and 18 healthy subjects were exposed in vitro to α-synuclein species. Cytokine/chemokine release was measured in the culture supernatant by Multiplex Elisa. The immune-phenotype was studied by FACS-flow cytometry. Correlation analysis was computed between immune parameters and parkinsonian motor and non-motor scales. We found that Parkinson’s disease patients exhibited a dysregulated PBMC-cytokine profile, which remained unaltered after exposure to α-synuclein species and correlated with both motor and non-motor severity, with a strong correlation observed with olfactory impairment. Exposure of PBMCs from healthy controls to α-synuclein monomer/oligomer increased the cytokine/chemokine release up to patient’s values. Moreover, the PBMCs immune phenotype differed between patients and controls and revealed a prominent association of the Mos profile with olfactory impairment, and of NK profile with constipation. Results suggest that a deranged PBMC-immune profile may reflect distinct clinical subtypes and would fit with the recent classification of Parkinson’s disease into peripheral-first versus brain-first phenotype.

**Supplementary Information:**

The online version contains supplementary material available at 10.1007/s00415-024-12554-3.

## Introduction

Parkinson’s disease (PD) is a complex neurodegenerative disorder characterized by a set of cardinal motor features variably associated with several non-motor symptoms (NMS) such as REM sleep behavior disorder, cognitive and mood changes, hyposmia, constipation, cardiovascular disturbances and others [1]. Clinical heterogeneity may reflect PD subtypes with different pathophysiology and pathological progression [2]. The neuropathological hallmark of PD is represented by deposit of Alpha-synuclein (αSyn), a monomeric protein that may also aggregate into toxic species and may be detectable in extracellular biofluids of patients with PD (PWP), including the cerebrospinal fluid (CSF), blood and saliva [3–6]. The initial αSyn pathology may occur in the brain or in the periphery, an observation that has led to the hypothesis that PD comprises two overall subtypes: a body-first subtype, in which αSyn pathology originates in the enteric nervous system and invades the CNS via the vagus nerve and sympathetic connectome; a brain-first subtype, in which pathology arises in the brain itself, most often in the limbic system or in the olfactory bulb. In this context, constipation has been considered as a prodromal NMS of PD with peripheral-onset, while hyposmia is a prodromal NMS linked to CNS involvement [7, 8].

Several earlier studies showing microgliosis [9, 10] and altered levels of brain’s cytokines [11, 12] have suggested that the immune system can play a pivotal role in PD pathology [13]. More recently, brain infiltrates of peripheral immune cells [14], peripheral inflammation and altered peripheral immune profile have been reported, indicating that immune response is systemically dysregulated [13]. Correlations between blood mononuclear cell (PBMCs) subpopulations or peripheral cytokines production and severity of motor/non-motor symptoms [15–28] also support the relevant role of central and peripheral immune changes in PD.

In the PD brain, αSyn is placed at the intersection between neurodegeneration and inflammatory responses. While neurotoxicity is mostly caused by αSyn aggregates [29–31], inflammatory response in microglia can be elicited by both monomeric and aggregated species [32, 33]. Moreover, αSyn may affect peripheral immune cells by stimulating cytokine overproduction, thereby contributing to immune activation [33–37]. These findings notwithstanding, several effects of αSyn monomers and oligomers on peripheral immune response and the relationship of immune changes with motor and non-motor clinical phenomenology remain to be definitely clarified. To investigate the role of monomeric and aggregated forms of α-syn in PD-associated inflammation, we analyzed a large panel of cytokines/chemokines as well as the immune cell profile in PBMCs isolated from PWP and healthy subjects (HS) upon stimulation with an amount of exogenous human αSyn monomer (αSynM) and oligomer (αSynO) described in the plasma of PD patients [38]. Correlations were drawn between PBMCs immune response and measures of motor and non-motor PD severity.

## Materials and methods

### Participants

PWP were enrolled at the outpatient Movement Disorder Clinic of the University of Cagliari. Diagnosis was made by a movement disorder expert according to the diagnostic criteria from the Movement Disorder Society [39]. Controls were HS attending the same center as caregivers or relatives of non-parkinsonian patients, with no history of PD or other neurodegenerative disorders. HS were clinically evaluated by the same physicians, and they were included in the study if both the neurological exam and the cognitive abilities were normal. None of the HS reported any of the typical prodromal NMSs of PD, such as REM sleep behavior disorder, olfactory deficit, constipation, and mood disorders. Exclusion criteria were atypical parkinsonism, dementia, immunological diseases requiring continuous immunomodulatory therapy, uncontrolled diabetes, recent vaccination against COVID-19 and infections occurring less than 4 weeks prior the recruitment. Motor severity was assessed by the modified Hoehn and Yahr (HY) scale [40] and Unified Parkinson’s Disease Rating Scale part III (UPDRS-III) Scale [41]. The burden of non-motor symptoms was assessed using the Non-Motor Symptoms Scale (NMSS) [42] that allows the identification of specific non-motor symptoms [43]. Total NMSS score and single items score were computed. Cognitive abilities were assessed by the Montreal Cognitive Assessment (MoCA) [44]. Data on current medications and disease duration were also collected. The levodopa equivalent daily dose (LEDD) was computed as previously reported [45]. The study was approved by the Local Ethical Committee (approval n. PG/2021/5461) and performed according to the Declaration of Helsinki. Participants were provided with an explanatory overview of the study and signed their consent to participate.

### Exogenous human α-synuclein species synthesis and purification

**αSynM.** αSynM was obtained through recombinant expression in E. coli using a pT7-7 plasmid, as previously described [29]. Protein was further purified by size exclusion chromatography (Hiload 26/60 Superdex 75 preparation grade, GE Healthcare, Little Chalfont, UK). Protein purity was assessed via SDS-PAGE, and protein concentrations determined spectrophotometrically.

**αSynO.** αSynO samples were prepared as previously described [29], starting from 6 mg of recombinant acetylated αSynM. αSynO samples were checked with circular dichroism and dynamic light scattering to conform with standard properties identified in our previous structural study [29]. Some samples were also tested for their cytotoxicity in neuronal cells using the MTT test [46]. After the purification procedure αSyn was tested for endotoxin contamination via the LAL (Limulus Amebocyte Lysate) assay (Kairosafe, Italy). The detection for bacterial endotoxin was constantly < 0.06 E.U./ml.

Fluorescent αSyn molecules were labelled with the AF647 dye (Invitrogen, Carlsbad, CA, USA) through ligation with the thiol moiety of Cys 122. Fluorescent oligomers were generated by mixing 90% unlabelled αSyn and 10% AF647-αSyn. The low ratio of labeled/unlabeled monomers and the position of the fluorescent probe in the C-terminal region, outside the structured oligomer core [29], ensured that no significant modifications to the oligomer properties were induced by the labelling protocol, as established by biophysical measurements.

### Samples collection and PBMCs isolation from whole blood

**Fig. 1:**
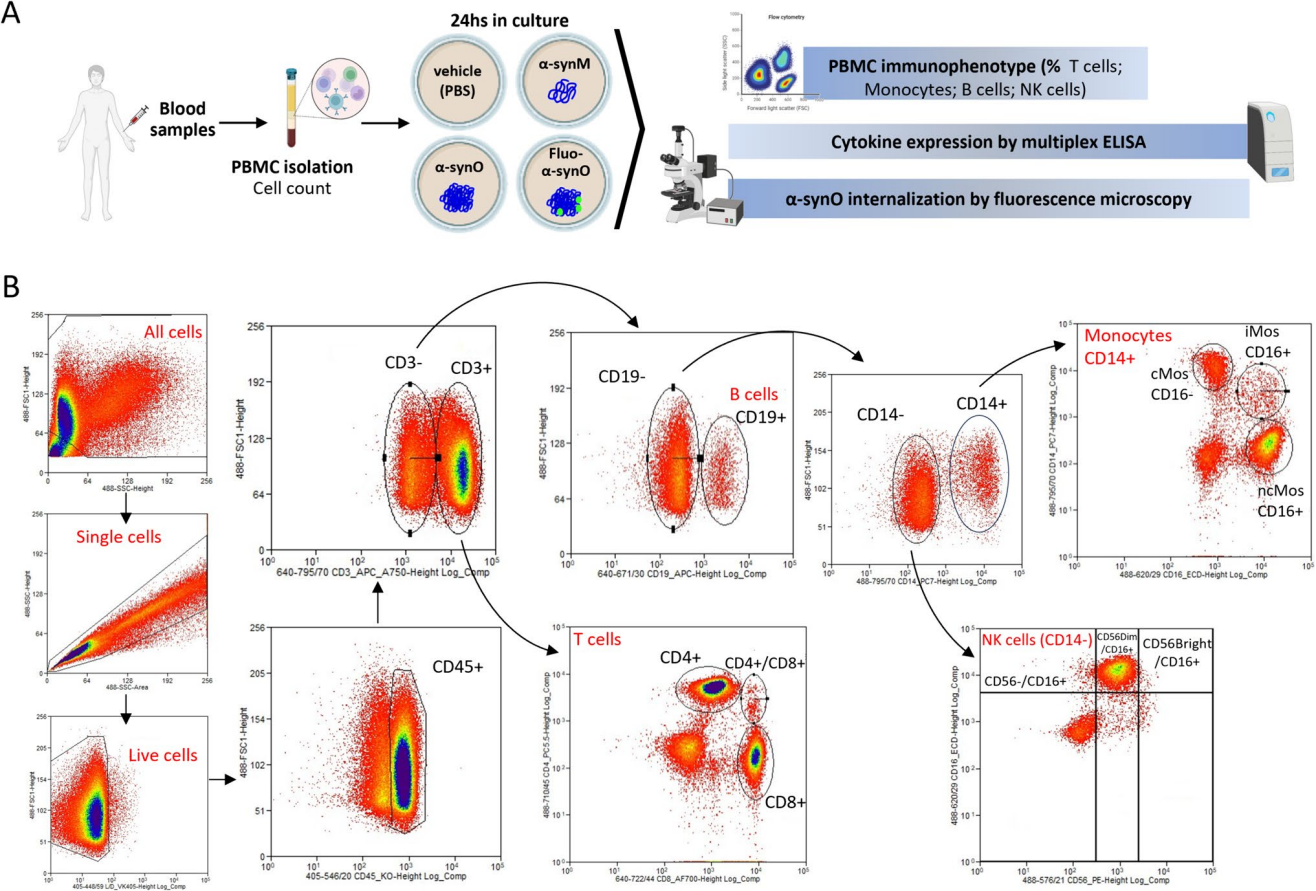
Experimental protocol and gating strategy. **A** Procedure and analyses of blood samples obtained from HS and PWP. Created using Biorender. **B** Gating strategy and analysed PBMCs population: T cells, B cells, Monocytes and NK cells with their respective markers

Figure 1A summarizes the experimental protocol. Fresh blood samples were collected and diluted (1:1) in Hanks’ Balanced Salt Solution (HBSS). PBMCs were isolated by density gradient media (Ficoll-Paque). The diluted blood was layered on top of an equal volume of Ficoll-Paque and centrifuged at 500 g and 20 °C for 30 min. The PBMC layer was collected and PBS-washed. Cells were counted by automatic Scepter^™^2.0 counter (Merck Millipore) and 8 × 10^5^/mL were cultured in RPMI supplemented with FBS 10–1% penicillin/streptomycin in 12-well plates, and treated for 24 h with 0.5 µM of αSynO or αSynM [29], or for 2 h with 0.5 µM of αSynO conjugated to FITC. The αSyn concentration was selected based on previous work reporting the αSynO content in peripheral blood of PWP [38].

### Cytokine and chemokine analysis *by multiplex* ELISA

Cytokines and chemokines release was assayed in the supernatant of PBMCs collected 24 h after αSynO/αSynM treatment. Cytokine & Chemokine Convenience 34-Plex Human ProcartaPlex™ Panel (EPXR340-12167–901, Thermo Fisher Scientific) was performed according to the manufacturer’s instructions. Analyte concentration was measured by Luminex MAGPIX (Luminex Corporation, Austin, TX) and data analyzed with xPONENT® software (Luminex Corporation, Austin, TX).

### Internalization of human α‐synuclein oligomers

PBMCs treated with FITC-conjugated αSynO were centrifuged at 500 g for 5 min, washed and resuspended in PBS. Cells were incubated with CD45-ECD (1:10, A07784, Beckman Coulter, Brea, CA) for 15 min at RT in the dark to visualize the membrane. The cell suspension was transferred on a slide and images of alive PBMCs were acquired (Olympus BX4, 40 × magnification).

### Immunophenotyping of PBMCs by FACS-flow cytometry

After incubation with αSynO/αSynM cells were collected, centrifuged at 500 g for 5 min, washed and resuspended in PBS. To exclude dead cells PBMCs were stained with the viability dye ViaKrome 405 (1:20, C36614, Beckman Coulter, Brea, CA). A classical FACS gating strategy was used to separate T cells, B cells, Mos and NK cells. To detect surface antigens cells were stained with a panel of specific monoclonal antibodies for 15 min at RT in the dark. The antibodies and relative concentrations were: CD45-KO (1:20, B36294), CD3-APC (1:20, A94680), CD4-PC5 (1:10, B16491), CD8-AF700 (1:10, B76279), CD19-APC (1:20, IM2470), CD14-PC7 (1:20, A22331), CD16-ECD (1:10, B49216), and CD56-PE (1:10, A07788), purchased from Beckman Coulter. After staining, cells were fixed with 1% PFA and analyzed with MoFlo Astrios EQs cell sorter (Beckman Coulter Inc, Brea, CA) with Summit version 6.3.1 software and 405, 488 and 642 nm lasers. Instrument compensation was set using the antibody capture beads kit VersaCom (B22804, Beckman Coulter, Brea, CA) following manufacturer’s instructions.

Figure 1B illustrates the gating strategy. Briefly, starting from CD45 + PBMCs, CD3 + T cells were separated into immune subpopulations based on single or double surface expression of CD4 and CD8 markers. CD3- cells expressing the surface marker CD19 were identified as B cells, while CD19- cells included Mos, NK cells and dendritic cells. Mos were further subdivided based on their expression of CD14 and CD16, into classical (cMos, CD14^high^/CD16^−^), intermediate (iMos, CD14^high^/CD16^high^), and non-classical (ncMos, CD14^low^/CD16^high^) [25, 47]. NK cells (CD14-) were separated based on the CD56 and CD16 expression, into immature NKs (imNK, CD56^bright^/CD16 +), mature NKs (mNK, CD56^dim^/CD16 +) and unconventional NKs (ucNK, CD56-/CD16 +) [48, 49].

### Statistical analysis

Statistical analysis was performed using Prism 8 (GraphPad Software, San Diego, CA, USA) and IBM SPSS Statistics for Macintosh, Version 29.0.2.0 (IBM Corp. in Armonk, NY). Data were expressed as means ± standard errors of the means (SEM), and analyzed by parametric or non-parametric tests (unpaired t test with Welch's correction and Mann–Whitney test) and one-way ANOVA followed by Tukey’s Multiple Comparison Test. In immunophenotype experiments, cell frequency data were presented as the percentage respect to each selected PBMCs subpopulation and to the total PBMCs population for each sample. The Spearman’s rank correlation coefficient with two-tailed p values was used to check for correlations between cell frequency in immunophenotype experiments and clinical scores, or between cytokines/chemokines concentration in multiplex ELISA experiments and clinical scores. To check the effect of multiple testing on single correlations, we used the original FDR (false discovery rate) method of Benjamini and Hochberg. A Quade nonparametric ANCOVA test considering age as a covariate, was used to verify whether age or disease duration may affect the differences observed between PD and HS internalization results.

## Results

Twenty-one PWP and 18 HS individuals participated into the study, (Table 1). The two groups were similar for sex (10 women and 11 men vs. 8 women and 10 men, *p* = 1) and age (70.5 ± 8.9 vs. 72.7 ± 7 years, *p* = 0.4).

**Table 1:** Demographic and clinical features of PD patients

	Parkinson’s Disease patients (n.21)
Mean age (years) of PD onset ± SD	70.5 ± 8.9
Mean PD duration (years) ± SD	5.3 ± 4.5
Mean HY staging ± SD	1.9 ± 0.7
Mean UPDRS- III score ± SD	25.3 ± 12.9
Mena LEDD (mg) ± SD	450.1 ± 422.1
Mean MoCA score ± SD	21.9 ± 6.2
NMSS (mean score ± SD): Total score Domain 1: Cardiovascular including falls Domain 2: Sleep/fatigue Domain 3: Mood/cognition Domain 4: Perceptual problems/hallucinations Domain 5: Attention/memory Domain 6: Gastrointestinal tract Domain 7: Urinary Domain 8: Sexual function Domain 9: Miscellaneous	53.5 ± 44.4 3.0 ± 3.8 6.25 ± 8.0 8.8 ± 15.1 0.55 ± 1.8 6.3 ± 9.2 6.6 ± 5.9 13.8 ± 13.7 3.8 ± 7.1 6.6 ± 6.8

Values are expressed as the mean + SD

*PD* Parkinson’s disease, *SD* standard deviation, *UPDRS* unified PD rating scale, *LEDD* levodopa equivalent daily dose, *HY* Hoehn and Yahr stage, *MoCA* montreal cognitive assessment, *NMSS* non-motor symptoms scale, *NA* not available

### Cytokine profile in culture medium from PBMCs

**Fig. 2:**
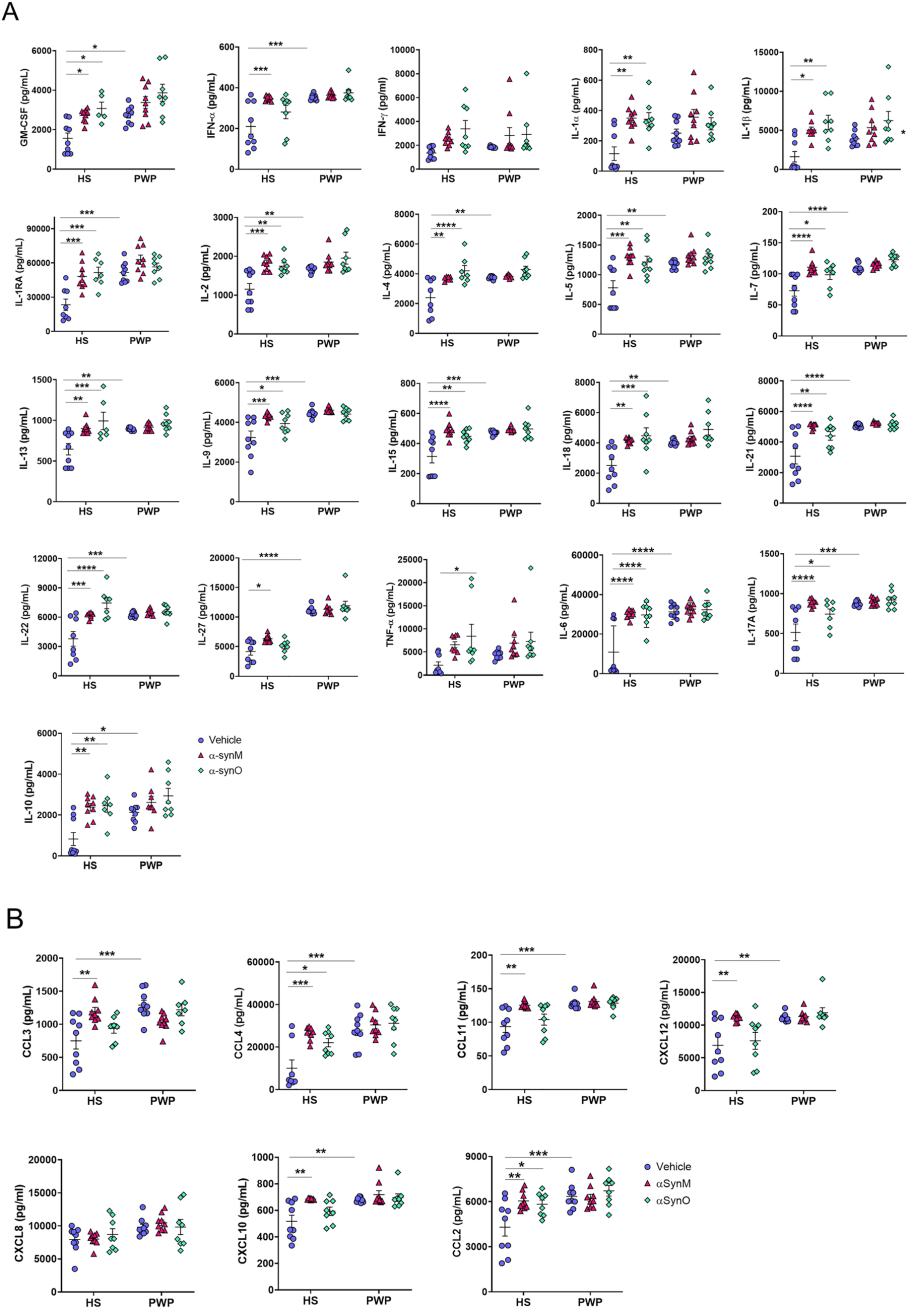
Cytokine (**A**) and chemokine (**B**) production at basal and after stimulation with human αSynM or αSynO. *p < 0.05, **p < 0.01, ***p < 0.001, ****p < 0.0001

Several inflammatory cytokines (i.e., IL-2, IL-6 and IL-17a), anti-inflammatory cytokines (i.e., IL-4, IL-10 and IL-13) (Fig. 2A) and chemokines (CCL3, CCL4 and CCL2) (Fig. 2B) were significantly higher expressed in the culture media of unstimulated PBMCs from PWP than HS. In vitro exposure to αSynM and αSynO did not modify the cytokine/chemokine release in PBMCs from PWP; by contrast, both αSyn species induced a potent inflammatory response in PBMCs from HS, with the release of cytokines and chemokines increasing to levels similar to those observed in PWP (Fig. 2A-B).

**Fig. 3:**
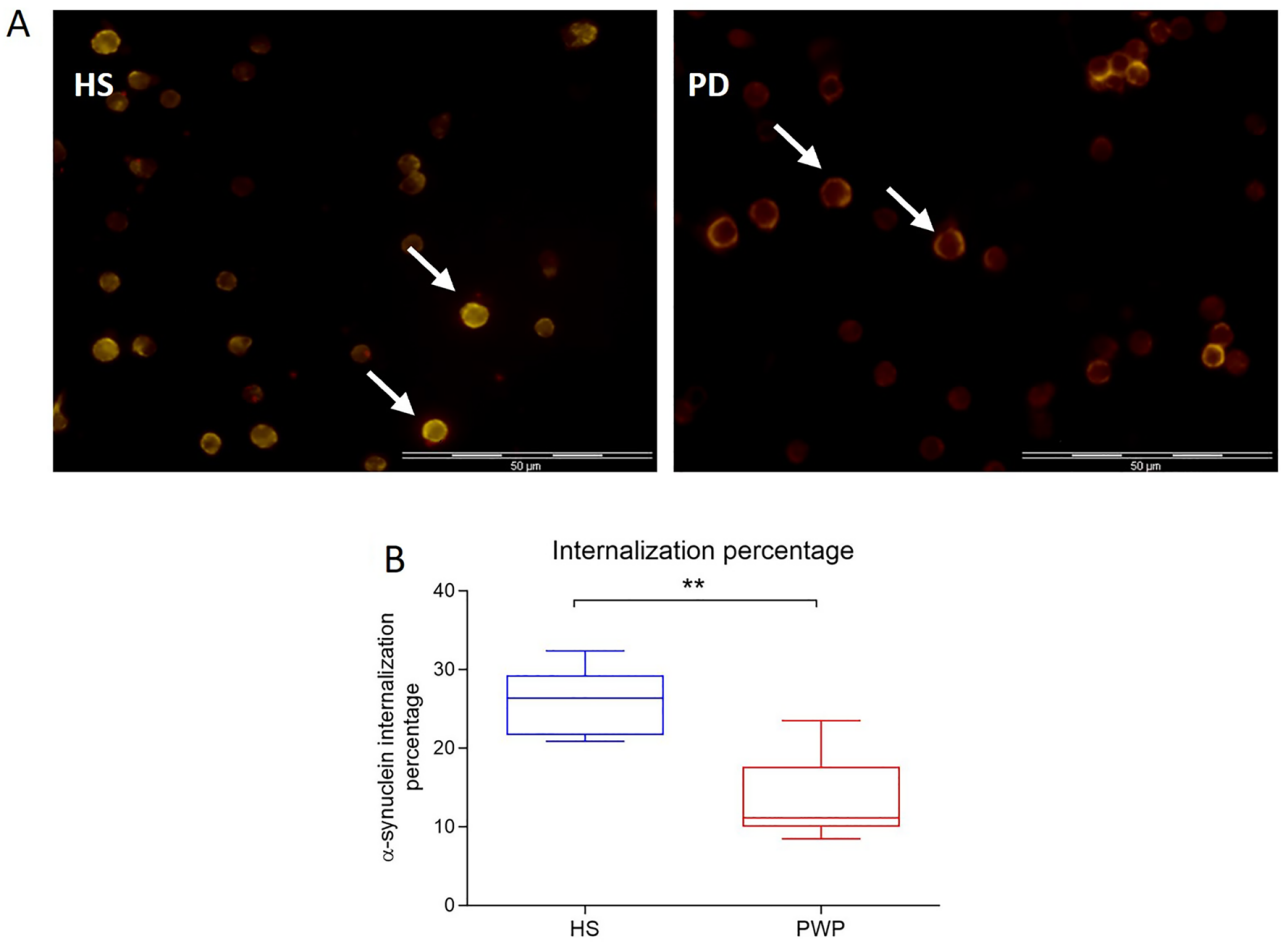
α‐synO internalization by PBMC in HS and PWP. **A** Fluorescence microscopy images showing α‐synO internalization in HS and PWP. **B** Internalization percentage in HS and PWP. Quade nonparametric ANCOVA test, **p < 0.001

Internalization of fluorescent FITC-conjugated αSynO was significantly lower in PBMCs from PWP than HS (10% vs. 26% of cells, *p* < 0.001 by Quade nonparametric ANCOVA test considering age as a covariate) (Fig. 3).

### Correlation of cytokine/chemokine concentrations and clinical measures

When we checked for possible relationships between cytokine/chemokine concentration from the entire panel and clinical measures, several significant correlations emerged (Table 2) The remaining correlations that failed to reach significance were not shown). Namely, IL-2, IL-5, IL-6 and IL-9 positively correlated with the Q28 parameter of the NMSS; IL-7 positively correlated with UPDRS-III score, total NMSS score, and the Q28 item; IL-18 positively correlated with UPDRS-III score, HY staging and the Q28 item; and the chemokine CXCL8 correlated positively with total NMSS score and the Q28 item.

**Table 2:** Correlations between cytokine/chemokine concentrations and clinical scales

	UPDRS Rho/*p* value	HY Rho/*p* value	NMSS Rho/*p* value	Q28 (Olfactory deficit) Rho/*p* value
IL-2	ns	ns	ns	0.94/0.002
IL-5	ns	ns	ns	0.84/0.014
IL-6	ns	ns	ns	0.90/0.005
IL-7	0.76/0.024	ns	0.69/0.048	0.80/0.025
IL-9	ns	ns	ns	0.85/0.014
IL-18	0.84/0.006	0.70/0.04	ns	0.78/0.03
CCL11	0.56/0.039	ns	ns	ns
CXCL8	ns	ns	0.57 / 0.008	0.90/0.005

Correlations (Spearman’s Rank correlation coefficient) between cytokine expression and clinical scales. Correlations that failed to reach significance are not shown

*ns* not significant, *UPDRS* unified PD rating scale, *HY* Hoehn and Yahr stage, *NMSS* non-motor symptoms scale

### PBMC immunophenotype

PBMCs immunophenotyping by FACS-flow cytometry yielded similar percentage of viable cells in PWP and HS (98.4 ± 1.10 vs. 97.2 ± 2.7, percent of total isolated PBMCs). Although the small number of subjects involved, the HS and PWP groups were matched for sex, and the sex effect on immune profile was evaluated. Since we did not find any significant sex effect on the immune profile, sexes were merged in graphs.

#### Monocytes

**Fig. 4:**
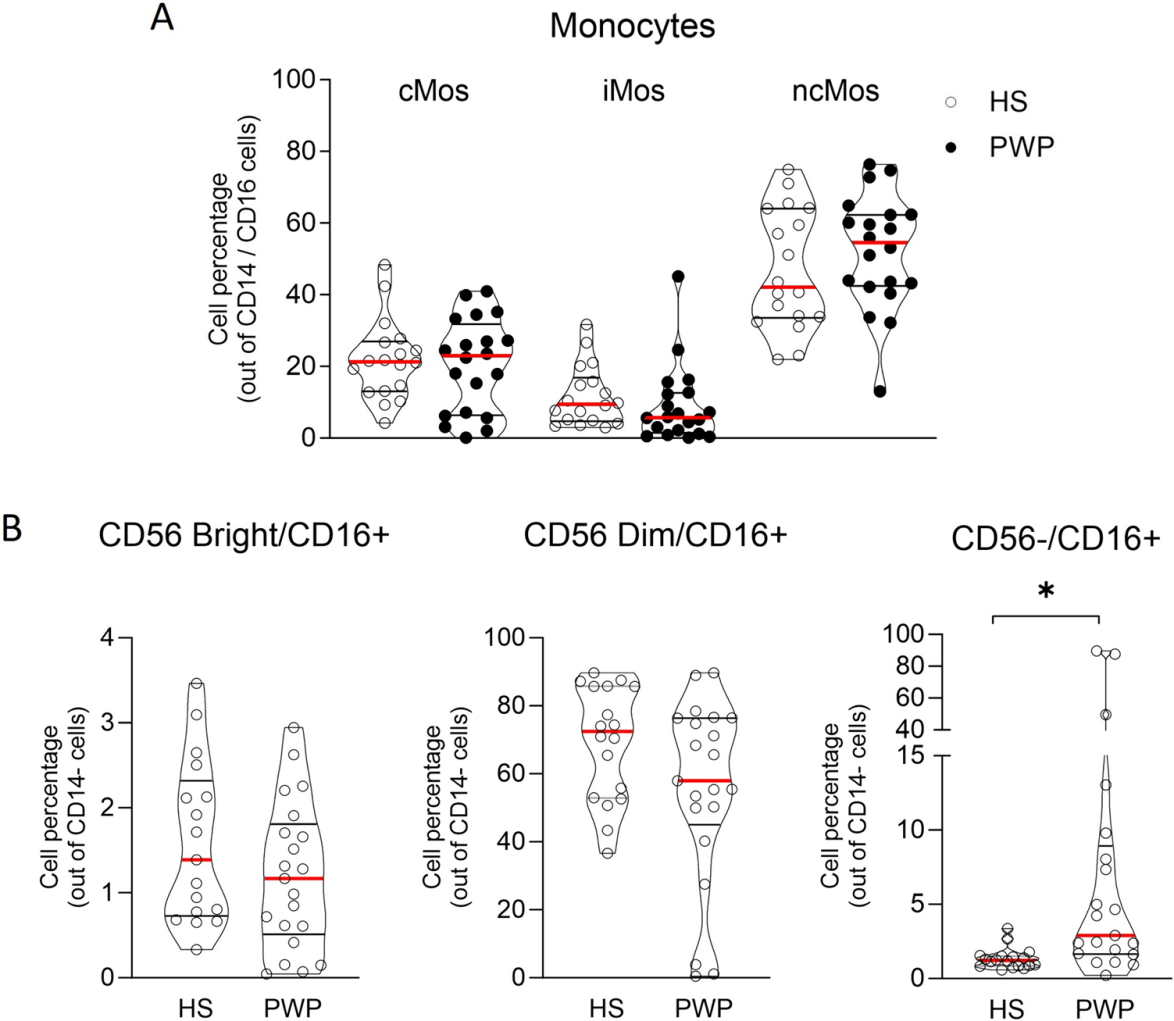
**A** Frequency of classical (cMos), intermediate (iMos) and non-classical (ncMos) monocytes out of CD14/CD16 subpopulation in HS and PWP. **B** Frequency of imNK (CD56 Bright/CD16 +), mNK (CD56Dim/CD16 +), ucNK (CD56-/CD16 +) in HS and PWP. T test with Welch’s correction, *p < 0.05

The percentage of Mos out of CD45 + PBMCs was similar in PWP and HS (supplementary Table 1). Three Mos subpopulations—cMos, iMos and ncMos—were identified according to CD14 and CD16 expressions (Fig. 4A). The frequency of the three subpopulations was comparable in PWP and HS (Fig. 4A). The Mos profile was not affected by treatment with αSynM or αSynO neither in PWP nor in HS (supplementary Table 1).

#### NKs

NK subpopulations were identified based on CD16 and CD56 expression (Fig. 4B and supplementary Fig. 1). The NK cell percentage out of CD45 + PBMCs was similar in PWP and HS (supplementary Table 1). As expected, the imNKs (CD56 Bright/CD16 +) represented the less frequent subpopulation and were similarly represented in PWP and HS. mNKs (CD56Dim/CD16 +) were the most frequent subpopulation and displayed a tendency to decrease in PWP (*p* = 0.067) (Fig. 4B). Finally, we observed a third NK subpopulation (CD56-/CD16 +), classified as unconventional NKs (ucNKs) based on previous description (Fig. 4B and supplementary Fig. 1) [48]. ucNKs were highly frequent in PWP but nearly absent in HS (*p* < 0.05) (Fig. 4B). Stimulation with αSynM or αSynO did not change the NK subpopulations frequency (supplementary Table 1).

#### T cells and B cells

The percentage of T cells out of CD45 + PBMCs was similar in PWP and HS (supplementary Table 1). Out of the total CD3 + T cell population, CD8 + cells tend to decrease, while CD4 + and double-positive (CD4 + CD8 +) cells tend to increase in PWP (supplementary Fig. 2) as previously reported [22]. Frequency of B cells showed a trending decrease in PWP (supplementary Fig. 2). Stimulation with αSynM or αSynO did not change the frequency of T or B cells (supplementary Table 1).

### Correlation of PBMC immunophenotype and clinical measures

When we checked for possible relationships between immune cell subpopulations and motor and non-motor symptoms, several significant correlations emerged.

**Fig. 5:**
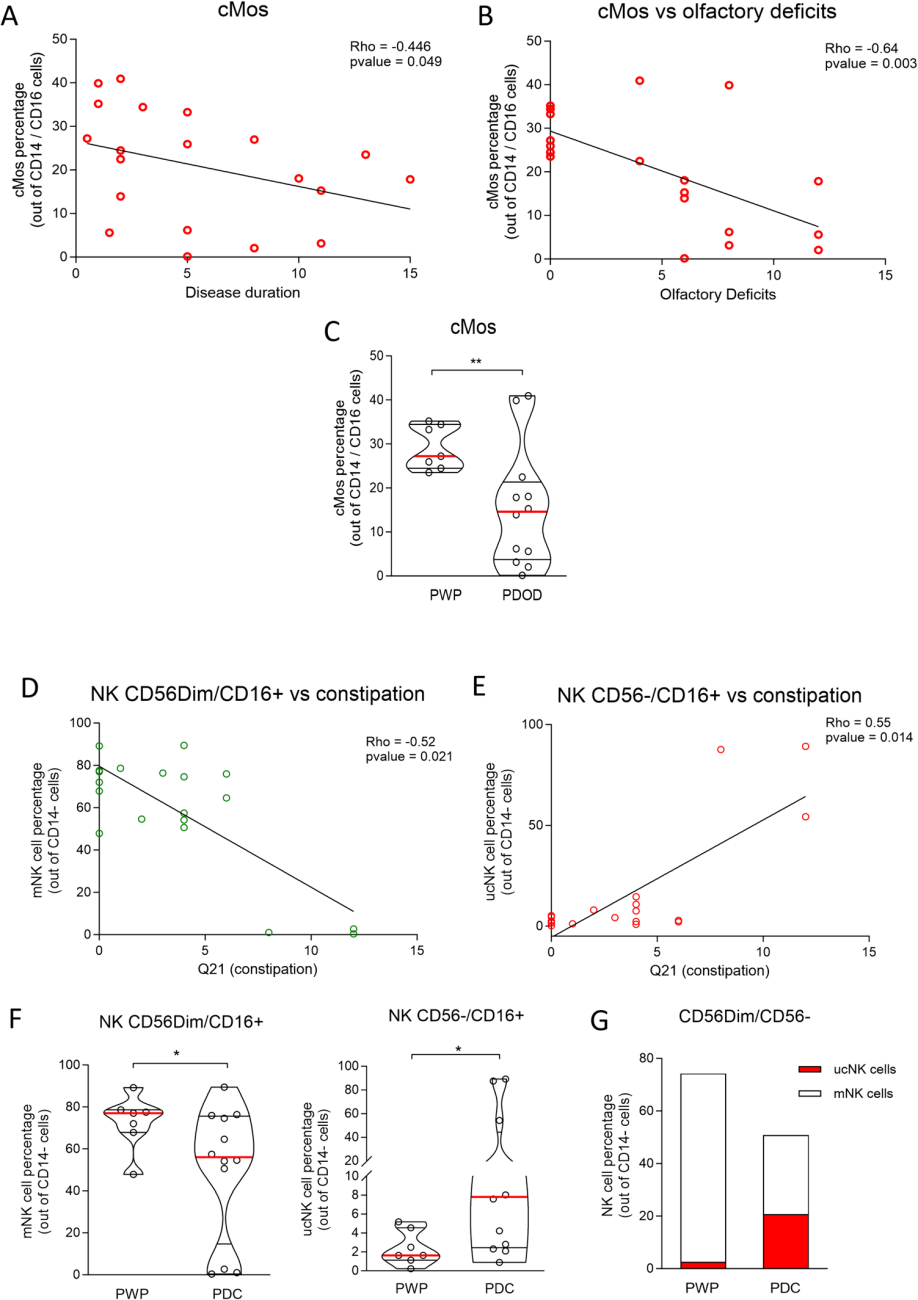
Spearman correlation between (**A**) cMos and disease duration and (**B**) cMos and Q28 item limited to olfactory deficit. **C** cMos frequency in PWP stratified for olfactory deficit. T test with Welch’s correction, *p < 0.05; **p < 0.01. Spearman correlation between (**D**) mNKs (CD16 + /CD56dim) or (**E**) ucNKs (CD16 + /CD56-) and the Q21 item for constipation. **F** mNKs and ucNKs frequency in PWP stratified for constipation. **G** mNK/ucNK ratio in PWP stratified for constipation. T test with Welch’s correction, *p < 0.05. PWP: patients with Parkinson’s disease; *PDOD* Parkinson’s disease patients with olfactory deficits, *PDC* Parkinson’s disease patients with constipation

An inverse correlation was found between cMos frequency and disease duration (Rho = − 0.446; *p* = 0.049) (Fig. 5A and supplementary Table 2), and between cMos frequency and the NMSS item “olfactory deficits” (Q28 in NMSS) (Rho = − 0.64; *p* = 0.003) (Fig. 5B and supplementary Table 2). The remaining correlations not reaching statistical significance were not shown. When patients were stratified by the NMSS Q28 item (presence of olfactory symptoms), the frequency of cMos was lower in patients reporting olfactory deficits (*p* < 0.01) (Fig. 5C). No other correlation between cMos frequency and other NMSS items could be detected.

The NK frequency did not correlate with any motor item nor with disease duration. However, there was an inverse correlation between mNK frequency and the NMSS Q21 item (constipation) (Rho = − 0.52, *p* value = 0.021) (Fig. 5D and supplementary Table 2) and a positive correlation between ucNKs and the NMSS Q21 item (Rho = 0.55, *p* value = 0.014) (Fig. 5E and supplementary Table 2. Remaining correlations that failed to reach significance were not shown). When patients were stratified by the NMSS Q21 item, the frequency of mNKs was lower, and the frequency of ucNKs was higher, in patients reporting constipation (*p* < 0.01) (Fig. 5F). Accordingly, the mNK/ucNK ratio decreased in patients reporting constipation (Fig. 5G) (*p* < 0.05). Finally, inflammatory cytokines IL-6, IL-9, IL-13, IL-21, as well the chemokine CCL4 positively correlated with ucNK frequency (*p* < 0.05; see supplementary Table 3 for details on Rho/p values).

## Discussion

This study analyzed the inflammatory profile and phenotype of PBMCs isolated from PWP and healthy individuals, before and after stimulation with αSynM and αSynO. PBMCs from PWP displayed a marked cytokine/chemokine inflammatory profile in the absence of exogenous αSyn stimulation, which correlated with the UPDRS-III score and NMSS total score. Stratifying by single NMSS items yielded a significant correlation with constipation. Stimulation with αSynM and αSynO could not further modify the inflammatory profile of PBMCs from PWP but raised the inflammatory response of PBMC from HS to levels comparable to those observed in unstimulated PBMCs from PWP. We also observed a reduced capacity of PBMCs from PWP to phagocytose αSyn in vitro. The PBMCs immune phenotype differed between PWP and HS. Mos correlated significantly with olfactory impairment, NKs correlated with constipation. Accordingly, stratification of patients by olfactory impairment or constipation revealed significant differences in the frequency of Mos and NK subpopulations, respectively.

The release of higher amounts of pro-inflammatory and anti-inflammatory cytokines and chemokines by unstimulated PBMCs from PWP extends previous reports on PBMCs or serum reporting variable results [11, 12, 17, 50–53]. The present findings supported a dysregulated peripheral immune response in PD and the possible recruitment of monocytes from periphery to the brain. Among the 30 cytokines and chemokines analyzed, some of them correlated with disease severity as assessed by UPDRS-III score (IL-7, IL-18 and CCL11) and with NMSS total score (IL-7 and CXCL8). Of note, cytokines IL-6, IL-2, IL-5, IL-7, IL-9, IL-18 and the chemokine CXCL8/IL-8 significantly correlated with the NMSS Q28 item (indicating olfactory deficit) but not with other NMSS items. This is a novel information that adds to a few previous studies exploring the association of peripheral cytokines and chemokines with motor and cognitive symptoms [15–17, 51, 52, 54–57]. The herein highlighted relationship between cytokine profile and olfactory impairment, an early sign that typically precedes cardinal PD motor signs, supports a contribution of peripheral inflammation to the pathophysiology of PD [13] and raises the possibility, to be explored, that measuring peripheral inflammatory species would contribute to diagnose prodromal PD.

The baseline cytokine/chemokine profile in PWP could not be modified by a stimulation with oligomeric αSyn at a concentration comparable to that described in the plasma of PD patients [38]. Instead, αSyn stimulation of PBMCs from HS raised the cytokine response to the same qualitative/quantitative level observed in unstimulated PBMCs from PWP. Although the present study was not designed to investigate dose-dependent responses, results may suggest that PBMCs collected from PWP were already highly activated. Moreover, results showed that both species of αSyn were inflammatory triggers for immune cells. This observation fits into the ongoing debate on differential toxicity of αSyn strains. While a number of studies have demonstrated that direct αSyn toxicity against neurons is structure-dependent [29, 58, 59], results were mixed about the inflammatory potential of monomeric and aggregated αSyn species against microglia and PBMCs [31, 37, 60]. This inconsistency could be related to the various degrees of toxicity displayed by diversely aggregated αSyn species used in these studies. Our results are in line with the previous reports showing that monomer and aggregated αSyn elicit a microglial response [37], and that both species bind to TLR2 and activate the downstream pathway [33]. Despite the quite different conformational properties of monomeric and oligomeric αSyn, the TLR2 has the capability to recognize a wide range of structurally unrelated PAMPs and DAMPs. The C-terminal and the N-terminal domains are exposed in oligomeric αSyn, and readily accessible in the unstructured monomeric state, thus potentially providing common interaction sites for TLR2. Therefore, unlike neurons peripheral leukocytes were similarly activated by oligomeric and monomeric αSyn species. Our observation was strengthened by the use of well-characterized human αSyn oligomers which are kinetically trapped in a toxic conformation and highly homogeneous in size and structural properties [29]. The purity of these oligomers previously enabled the characterization of their toxicity mechanisms in vitro and in vivo against neurons and glial cells [61, 62].

The reduced capability of PBMCs from PWP to phagocytose αSyn in vitro was consistent with studies showing a decreased expression of TLR4 in peripheral phagocytes from PWP [25], and studies indicating a reduced capacity of αSyn clearance by glial cells in PD models [61, 63–65].

Although the percentage of Mos subpopulations did not differ in PBMCs from PWP and HS, cMos inversely correlated with disease duration and the NMSS item Q28 “olfactory deficits”. This is a further novel information that strengthened the aforementioned association between several cytokines/chemokines and olfactory deficit. Previous studies have shown that Mos are highly dynamic and stage-dependent in PD, showing an increase of cMos in the early disease stage but not at later stages, in line with our results [24, 25]. Moreover, a negative correlation was reported between frequency of cMos and measures of cognitive impairment in PWP [25] supporting a critical involvement of Mos dysregulation in the brain pathology. Our finding of an overproduction of monocyte-chemoattractant chemokines by PBMC from PWP, supports the possibility that chemokines would drive Mos to migrate from blood to the inflamed brain tissue. Other studies reported varying results on Mos profile, likely reflecting the disease stage analyzed and the gating strategy applied [52, 66].

The few studies that have examined changes in peripheral NK frequency in PWP reported uneven results [25, 26, 28, 67–70], probably due to pronounced differences in the markers used to identify these cells. Our characterization of NK profile based on classical CD56/CD16 expression [71] yielded the identification of three subpopulations, namely immature NKs, terminally mature and cytotoxic NKs, and a third subpopulation categorized as unconventional NKs*.* Typically, unconventional NKs increase during viral infections or autoimmune diseases [48, 49, 72], with their expansion being associated with a decrease in the mature subpopulation and regulated by cytokines [48, 49]. Consistently, we found a positive correlation between cytokines production in PWP and the frequency of ucNKs. Notably, the unconventional NK subset was significantly more represented among PWP while the mature subset was significantly more present in HS. Moreover, this is the first report describing an inverse specific correlation of both mature and unconventional NKs with constipation. Hence, mNK and ucNKs were respectively low and highly represented in a subgroup of PWP reporting constipation, and inversely correlated with this symptom. This report adds to the few studies that investigated NK cell frequency in relation with other PD clinical features [73–75]. Interestingly, multiple lines of evidence suggest a relationship between viral or bacterial exposures, alterations in gut microbiota, and the increased risk of developing PD [13]. Our finding further supports the specific involvement of the NK population in PD phenotypes with gut disturbances.

The clinical heterogeneity of PD may reflect subtypes with distinct pathophysiology and progression, namely peripheral-first versus brain-first phenotype, whose differential diagnosis would benefit of specific biological parameters. While the body-first subtype implies that pathology originates in periphery, including the enteric nervous system, and subsequently invades the CNS, the brain-first subtype implies that pathology arises in the brain itself, most often in the limbic system or in the olfactory bulb. In this context, constipation has been proposed as a prodromal symptom reflecting peripheral-onset, while prodromal hyposmia may reflect CNS onset [7, 8]. The present finding of correlations between specific immune cell populations and specific NMS of PD well fits into this scenario and may aid the early differential diagnosis of peripheral versus brain-onset phenotypes, although larger population-based studies are warranted to consolidate our findings. Hence, the NK profile was mostly affected by constipation and may support the early recognition of body-first PD phenotypes, while the Mos profile changed in relation to olfactory deficits and severity/duration of motor symptoms, supporting recognition of brain-first phenotypes.

This study has strengths and limitations. The research was conducted on a limited number of PWP, and a selection bias cannot be ruled out. Nevertheless, the inclusion of consecutive patients throughout the study period and their diagnosis by movement disorder specialists of the same center, following the same study protocol, provided a sample reflective of the typical PD population. We believe that PD patients in our study were relatively homogeneous in terms of disease stage. Indeed, this is supported by the low variability in the HY stage and the UPDRS scores, which are values of a consistent disease severity across participants. Our control group was composed solely of HS, whereas others also considered patients with a variety of neurological conditions mimicking PD, such as dementia with Lewy Bodies and multiple system atrophy. To assess NMDS, we used the NMSS, a widely used tool that is based on patient self-reporting and may thus be influenced by the individual's perception and understanding of their symptoms. Expanding our results with specific questionnaires, such as the semi-objective olfactory evaluation with the Sniffin' Sticks test or the University of Pennsylvania Smell Identification Test would be needed.

PBMCs were freshly analyzed to avoid any freeze and thaw cycle which may affect phenotyping and the relative subpopulation percentage. To avoid any methodological bias and allow data comparison, all samples were immediately processed after collection and equally isolated by density gradient media. Although it has been reported that the expression of PBMC cell surface markers differ across cell isolation procedures and upon cell culture respect to direct *ex-vivo* measurement [76, 77], cell culture was mandatory to test the effect of αSyn monomer and oligomer. A similar protocol was used in the previous studies involving PBMC cultures [37].

Importantly, we took advantage of structurally characterized and highly homogeneous human αSyn oligomers to stimulate PBMCs. In addition, the multiplex Elisa test used in the present study enabled the assessment of a wide range of cytokines and chemokines in a single well and in the same sample aliquot, thus overcoming caveats arising from different individual cytokine assays comparison. Owing to the small size of the sample and the low statistical power, correlation analysis for most NMSS items yielded inconclusive results. Nevertheless, the significance level reached by some correlation analyses including the NMSS Q28 and Q21 items, despite of low study power, would suggest a greater magnitude of the association with some immune parameters for the Q28 or Q21 NMSS items than for the other items.

## Conclusion

Our findings provided new insights on immune response in PWP. While confirming and extending several deviations in peripheral immune profile of PWP, the significant correlations between some immune and clinical parameters unveil an uneven behavior of immune subpopulations in relation with specific NMS. The prevalent association of the Mos profile with olfactory impairment and the association of NK profile with a peripheral NMS such as constipation would fit with the recent classification of PD into subtypes with different pathological onset, namely peripheral versus brain-first phenotype. In the context of the clinical heterogeneity of PD, measurement of peripheral immune parameters may aid to differentiate peripheral versus brain-onset phenotypes. Our findings also highlight the potential relevance of peripheral inflammatory parameters in delineating prodromal PD.

## Supplementary Information

Below is the link to the electronic supplementary material.

Supplementary file1 (PDF 209 KB)
