# Supplementary material for: Immune responses to oligomeric α-synuclein in Parkinson’s disease peripheral blood mononuclear cells
Source: J Neurol. 2024 Jul 10;271(9):5916–29. doi: 10.1007/s00415-024-12554-3 (PMC11377674; doi:10.1007/s00415-024-12554-3)
Supplement: Supplementary file 1 — Supplementary file1 (PDF 209 KB) [file 415_2024_12554_MOESM1_ESM.pdf]

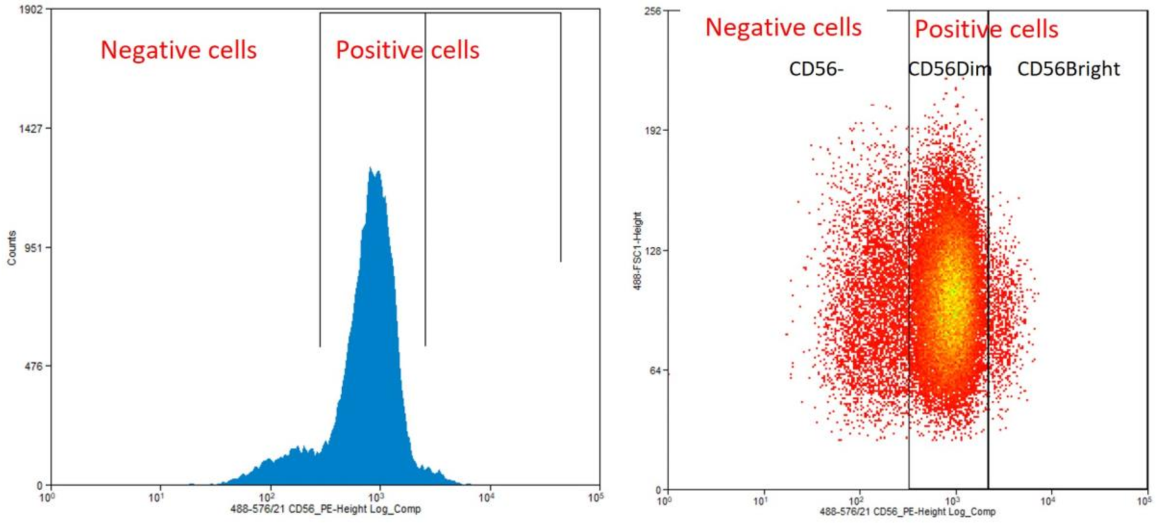

Fig. S1. Histogram and dot plot of CD56 expression for threshold setting.

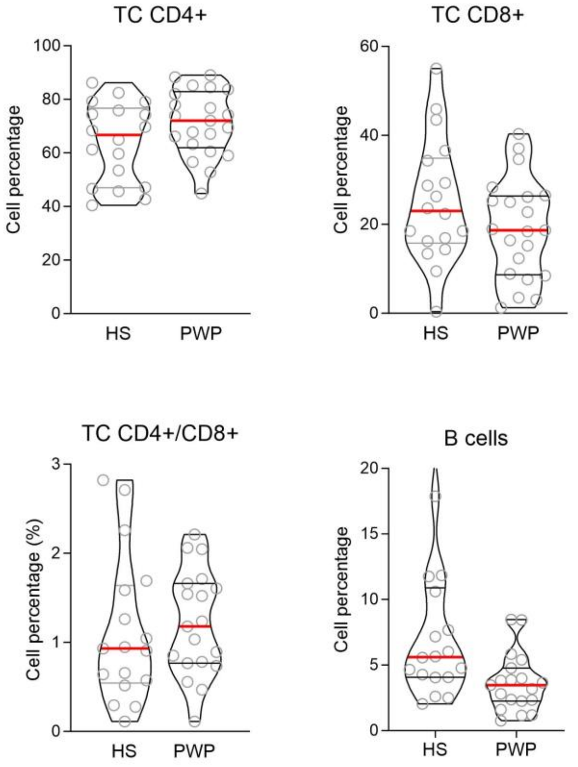

Fig. S2. T cells, B cells and CD19- cells in healthy subjects and Parkinson's disease donors. Frequency of CD4+, CD8+, CD4+ CD8+ T cells and CD19+ cells in HS and PWP.

PBMC populations	Cell percentage ± SD					
	HS			PWP		
	Vehicle	αSynM	αSynO	Vehicle	αSynM	αSynO
cMos	5.19 ± 3.1	5.9 ± 4.5	5.33 ± 3.5	4.25 ± 3.8	3.15 ± 3.3	4.46 ± 4.6
iMos	3.29 ± 3	0.98 ± 1	1.36 ± 1.5	2.1 ± 2.2	2.7 ± 2.8	2.41 ± 2.5
ncMos	13.4 ± 10.4	13.42 ± 10.2	14 ± 10.9	18 ± 8.3	18 ± 8.5	17.97 ± 8.3
NK cells	14.4 ± 10.7	14.57 ± 10.8	15.15 ± 11.1	18.01 ± 9.6	17.94 ± 9.7	17.86 ± 9.4
T cells	66.34 ± 13.7	67.69 ± 13.9	66.95 ± 13.8	56 ± 18.2	56 ± 17.7	55 ± 18.5
B cells	5.15 ± 2.6	5.32 ± 2.8	5.14 ± 2.5	3.14 ± 1.5	3.2 ± 1.6	3.2 ± 1.6

Table S1. Frequency of PBMC populations out of CD45+ cells at basal, αSynM and αSynO conditions.

	**Q21 (Constipation)**	**Q28 (Olfactory Deficits)**	**Disease duration**
	Rho / p value	Rho / p value	Rho / p value
**cMos**	0.16 / 0.517	-0.64 / 0.003**	-0.446 / 0.049*
**ucNKs**	0.55 / 0.014*	-0.21 / 0.383	-0.331 / 0.154
**mNKs**	-0.52 / 0.021*	0.28 / 0.243	0.247 / 0.293
**imNKs**	-0.47 / 0.043*	0.07 / 0.785	0.225 / 0.341

Table S2. Spearman's coefficient (Rho) of cMos / NK cell subpopulations and Q21 / Q28 clinical parameters and Disease duration. Rho: Spearman correlation coefficient; *$p < 0.05$, **$p < 0.01$.

	**Unconventional NKs**
	Rho / p value
**IL-6**	0.72 / 0.03*
**IL-9**	0.79 / 0.01*
**IL-13**	0.77 / 0.02*
**IL-21**	0.78 / 0.015*
**CCL4**	0.70 / 0.04*

Table S3. Significant correlations between cytokines and unconventional NKs at basal condition in PWP. Rho: Spearman correlation coefficient; *$p < 0.05$.
